# The Class III PI3K/Beclin-1 Autophagic Pathway Participates in the mmLDL-Induced Upregulation of ET_A_ Receptor in Mouse Mesenteric Arteries

**DOI:** 10.1155/2020/5070436

**Published:** 2020-04-01

**Authors:** Xi Xie, Chen Chen, Cang-Bao Xu, Jie Lin, Lei Cao, Gen Chen, Jie Li

**Affiliations:** ^1^The First People's Hospital of Chenzhou, Institute of Pharmacy and Pharmacology, University of South China, Hengyang, Hunan, China; ^2^Shaanxi Key Laboratory of Ischemic Cardiovascular Disease, Institute of Basic and Translational Medicine, Xi'an Medical University, Xi'an, China; ^3^Department of Pharmacology, School of Basic Medical Sciences, Xi'an Jiaotong University Health Science Center, Xi'an, Shaanxi, China

## Abstract

Minimally modified low-density lipoprotein (mmLDL) is a risk factor for cardiovascular diseases. The current study explored the effect of mmLDL on the endothelin type A (ETA) receptor in mouse mesenteric arteries *in vivo*, as well as the role of autophagy in this process. mmLDL was injected via the caudal vein, and the Class III PI3K autophagic pathway inhibitor 3-methyladenine (3-MA) was injected intraperitoneally. The animals were divided into physiological saline (NS), mmLDL, and mmLDL + 3-MA groups. The dose-effect curve of endothelin-1- (ET-1-) induced mesenteric artery contraction was measured using myography, while ET_A_ receptor mRNA expression was detected using real-time polymerase chain reactions, and the protein levels of the ET_A_ receptor, class III PI3K, Beclin-1, LC3 II/I, p62, NF-*κ*B, and p-NF-*κ*B were observed using Western blot analysis. mmLDL significantly strengthened ET-1-induced contraction (the *E*_max_ value increased from 184.87 ± 7.46% in the NS group to 319.91 ± 20.31% in the mmLDL group (*P* < 0.001), and the pEC_50_ value increased from 8.05 ± 0.05 to 9.11 ± 0.09 (*P* < 0.01). In addition to upregulating the protein levels of Class III PI3K, Beclin-1, and LC3 II/I and downregulating that of p62, mmLDL significantly increased the mRNA expression and protein level of the ET_A_ receptor and increased the protein level of p-NF-*κ*B. However, these effects were significantly inhibited by 3-MA. mmLDL activates autophagy via the Class III PI3K/Beclin-1 pathway and upregulates the ET_A_ receptor via the downstream NF-*κ*B pathway. Understanding the effect of mmLDL on the ET_A_ receptor and the underlying mechanisms may provide a new idea for the prevention and treatment of cardiovascular diseases.

## 1. Introduction

Autophagy is an evolutionally conserved metabolic process; as an important survival mechanism, it degrades and turns over organelles and cytoplasmic contents [1]. The autophagy process consists of the formation of bilayer-membrane autophagosomes, the phagocytosis of cytoplasmic substances, and the fusion of autophagosomes with lysosomes for degradation. The formation of autophagosomes depends on the assembly of Beclin-1- and Class III PI3K-containing phospholipid kinase signaling complexes. These complexes mediate the nucleation of the autophagosomal precursor and activate two ubiquitin-like binding pathways (Atg5-Atg12 and microtubule-associated protein light-chain 3-phospholipid conjugates) to promote separation and membrane expansion [2, 3].

The endothelin (ET) system is composed of ligands and receptors, and an imbalance in this system plays an important role in the pathogenesis of cardiovascular diseases. ET-1 serves as a highly active vasoconstrictor that activates the endothelin type A (ET_A_) and endothelin type B (ET_B_) receptors. ET-1 imbalance is associated with pathological conditions, such as arteriomesenteric ischemia reperfusion [4]. The ET_A_ receptor is highly expressed on the vascular smooth muscle cells (VSMCs) of the entire cardiovascular system, which mediates the constriction, proliferation, apoptosis, and fibrosis of the vessels [5]. In myocytes with the specific knockout of ET_A_ receptors, aging-induced myocardial hypertrophy and contractile dysfunction are improved, during which autophagy exerts an important role [6].

Minimally modified low-density lipoprotein (mmLDL) is a mildly oxidized low-density (oxLDL) lipoprotein in which some of the lipids are oxidized but that maintains an integral apolipoprotein B structure. mmLDL can be identified by LDL receptors other than macrophage scavenger receptors [7]. mmLDL induces the proinflammatory effect of macrophagocytes, which includes cytoskeletal rearrangement, macrophage pinocytosis, ROS production, foam cell formation, and decreased apoptotic cell phagocytosis [8]. mmLDL possesses multiple atherosclerosis-promoting features. For instance, mmLDL induces the adhesion of monocytes to endothelial cells to generate colony-stimulating factors, monocyte chemotactic protein-1, and tissue factors, which causes damage to endothelial cells and promotes oxLDL generation; additionally, mmLDL can activate multiple apoptotic signaling pathways in human coronary artery cells [9].

Here, for the first time, we explored the effect of mmLDL on arteriomesenteric ET_A_ receptors *in vivo* and the role of autophagy in this process. The results of this study may deepen the understanding of the vascular biological mechanisms of mmLDL.

## 2. Materials and Methods

### 2.1. mmLDL Preparation

The thiobarbituric acid-reactive substance (TBARS) method and agarose gel electrophoresis were used for examining differences between LDL, mmLDL, and oxLDL. According to the TBARS method, the oxidized apolipoprotein B, the cholesterol oxidation products, and the lipid peroxidation products in blood plasma are all able to react with thiobarbituric acids to form malondialdehyde-like structures, and the oxidation degree of LDL can subsequently be assessed based on the number of the structures. The natural concentration of the malondialdehyde-like structures of LDL is lower than 4 *μ*mol/g LDL, whereas the natural concentrations of mmLDL and oxLDL are between 5 *μ*mol/g LDL and 20 *μ*mol/g LDL and beyond 20 *μ*mol/g LDL, respectively. According to agarose gel electrophoresis, oxLDL has the highest level of oxidation with a large number of electrical negatives at a fast migration rate, which is followed by mmLDL and then LDL [10, 11].

Human LDL (0.2 mg/mL; Sigma, St. Louis, MO, USA) was applied to EDTA-free phosphate-buffered saline (PBS) containing 1 *μ*mol/L FeSO_4_. After a 96-hour dialysis at 4°C, 0.25 mM EDTA was used to terminate the oxidizing reaction for mmLDL [10, 11]. The mmLDL was concentrated to 1 mg/mL. The degree of mmLDL oxidation was determined with TBARS, and the result showed that the concentration of malondialdehyde-like structures was 10.36 ± 1.15 *μ*mol/g LDL. Electrophoresis (0.8% agarose gel) showed that the mobility of mmLDL was 1.66–1.70 times that of LDL. The prepared mmLDL was stored away from light at 4°C and used within 2 weeks.

### 2.2. Animals and Grouping

SPF-level ICR mice of both sexes, with age ranging from 8 weeks to 11 weeks and a weight of 20–24 g, were purchased from the animal center of Xi'an Jiaotong University, China. The animals had free access to water and food and were divided for different purposes, with 10 animals in each group. The animal treatment protocols were approved by the Institute of Animal Ethics of Xi'an Jiaotong University.

To investigate the effect of mmLDL on mouse arteriomesenteric ET_A_ receptors, the mmLDL group received 8 injections of 1 mg/kg mmLDL via the caudal vein at an injection interval of 12 h. At 96 h, the animals were killed via cervical dislocation after eyeball blood was drawn; then, the mesenteric arteries were collected for subsequent experiments [11–13]. The normal saline (NS) group and the LDL group were given the same dosage of physiological saline and LDL, respectively.

To explore the effect of the Class III PI3K/Beclin-1 pathway, an intraperitoneal injection of the Class III PI3K inhibitor 3-methyladenine (3-MA; MedChemExpress, Shanghai, China) was simultaneously administered to the animals. To determine the optimal dose of 3-MA, 5, 15, and 25 mg/kg groups were designed. The required concentration of 3-MA was prepared with double distilled water. The injection was performed 2 h before the first injection of mmLDL every day, once per day. At 96 h, the mice were killed using the cervical dislocation method, and 3-MA was not given within 2 h before the sacrifice [12]. The optimal 3-MA dose was determined based on ET_A_ receptor-mediated vasoconstriction curves.

### 2.3. Detection of Vasoconstriction Function

After a mouse was killed, the tissue containing the mesenteric arteries was immediately collected and then immersed in a Na^+^-Krebs solution (containing 15 mM NaHCO_3_, 119 mM NaCl, 1.2 mM MgCl_2_, 1.2 mM NaH_2_PO_4_, 1.5 mM dehydrated CaCl_2_, 4.6 mM KCl 4.6, and 5.5 mM glucose) at 4°C. The surrounding tissue was isolated under a microscope, and a 10 s intravascular perfusion was performed with 0.1% Triton x-100. The vascular endothelium was removed. The sample was washed with buffer for 10 s, and then 20 *μ*M 5-HT was used for a preshrinking test. When the acetylcholine- (ACh-; 10 *μ*M) induced diastolic rate was lower than 10%, the endothelium was considered successfully removed.

The arteries were cut into rings with a length of 1-2 mm. Two metal wires with a diameter of 40 *μ*m were inserted through the rings, with one connected to a load tension readjustment equipment and the other connected to a myographer (Danish Myo Technology A/S, DMT, Denmark). The rings were placed into a thermostatic tank (37°C) full of Na^+^-Krebs solution. To achieve oxygen saturation, mixed gas containing 95% O_2_ and 5% CO_2_ was continuously ventilated. The pH value was maintained at approximately 7.4, and the resting tension was maintained at 1.5 mN. The Krebs solution was replaced every 20 min, with an equilibrium time of 1.5 h. K^+^-Krebs solution (containing 15 mM NaHCO_3_, 1.2 mM NaH_2_PO_4_, 1.5 mM CaCl_2_, 63.5 mM KCl, 1.2 mM MgCl_2_, 60 mM NaCl, and 5.5 mM glucose) was applied to detect the activity of the rings. The arterial rings were rinsed three times. The abovementioned procedures were repeated. When the contractile amplitudes caused by the two applications of K^+^-Krebs liquid were both higher than 1 mN and the difference between the contractile amplitudes was within 10%, subsequent experiments were carried out. To investigate ET_A_ receptor-mediated contractile function, the vascular rings were incubated with the ET_B_ receptor antagonist BQ-788 at 0.1 *μ*M (Sigma, St. Louis, MO, USA) for 30 min. Then, ET-1 (Enzo Biochem, Shanghai, China) (ET-1 is the agonist of the ET_A_ and ET_B_ receptors, and ET_A_ receptor-specific agonists have not been discovered) at 10^−11^–10^−7^ M was added using the concentration accumulation method, and the dose-effect curve of ET_A_ receptor-mediated vasoconstriction was obtained [14, 15]. The pretest showed that the simultaneous intraperitoneal injection of polymyxin B during the caudal vein injection of mmLDL did not affect the effect of mmLDL, and therefore, the possibility of endotoxic interference was excluded.

### 2.4. Real-Time Polymerase Chain Reaction (RT-PCR)

Total RNA was extracted using the RNA extraction kit (Proteintech Biotechnology, USA), and the procedures were performed in strict accordance with the kit instructions. The absorbance at 260 nm and 280 nm was detected to determine the concentration and purity of the total RNA. Reverse transcription was performed for cDNA (Perkin-Elmer Applied Biosystems, USA). A fluorescent quantitative PCR instrument (LightCycler 480 II system) was used for RT-PCR. A SYBR Green kit was utilized for gene amplification, and cDNA was used as the template. A control group was established, and the procedures were the same as those performed for the experimental group except for the addition of cDNA. The forward and reverse primers of the ET_A_ receptor were 5′-TGCCTCTGTTGCTGTT-3′ and 5′-TGTGGTTGTTCTGCTCTTGG-3′, respectively, and those of the housekeeping gene GAPDH were 5′-TCAACGGCACAGTCAAG-3′ and 5′-ACTTCCACGACATACTCAGC-3′, respectively. The reaction system was 25 *μ*L, and the conditions included 95°C for 5 min followed by 40 cycles of 94°C for 20 s, 55°C for 20 s, and 72°C for 30 s. Separation efficiency curves were used for the analysis of the specificity of the PCR products, and the cycle threshold (*C*_T_) method was used for quantitative mRNA analysis. The *C*_T_ value of GAPDH was used as the internal reference, and the relative mRNA expression of the ET_A_ receptor was calculated based on that value.

### 2.5. Western Blotting Analysis

Each sample consisted of two mesenteric arteries from the same group. RIPA lysis buffer containing proteinase inhibitors was added, and homogenate was prepared. A BCA kit was used for protein quantification. The extracted protein was subjected to SDS-PAGE followed by isolation and PVDF membrane transfer. Evaporated milk (5%; prepared with T-TBS buffer) was applied for sample blocking. The primary antibodies included ET_A_ receptor antibody (dilution, 1 : 500; GeneTex, Inc., USA), Class II PI3K antibody (1 : 1000; Cell Signaling Technology), p-NF-*κ*B p65 antibody (1 : 1000; Cell Signaling Technology), Beclin-1 antibody (1 : 3000; Proteintech Biotechnology, Inc., USA), LC3 antibody (1 : 500; Proteintech Biotechnology), p62 antibody (1 : 3000; Proteintech Biotechnology), NF-*κ*B antibody (1 : 3000; Proteintech Biotechnology), and GAPDH antibody (1 : 1000; Proteintech Biotechnology), and they were prepared with PBS containing 2% bovine serum albumin as the diluent. The secondary antibodies were peroxidase-labeled goat anti-rabbit IgG and goat anti-mouse IgG (both, 1 : 5000). Images were taken with Image Gauge Ver. 4.0 (Fuji Photo Film Co., Ltd, Japan) for optical density analysis. Grayscale self-correction was performed. The level of the target protein is presented as the ratio between the band grayscale of the target gene and that of the housekeeping gene.

### 2.6. Serum oxLDL Determination

After the animals were sacrificed, the eyeballs were excised, and blood was collected. The blood sample was coagulated at room temperature for 30 min. The sample was centrifuged at 845 ×g for 30 min at 4°C, and the supernatant was collected for analysis. The concentration of serum oxLDL was determined using enzyme-linked immunosorbent assays (ELISAs). The procedures were performed in strict accordance with the kit instructions (Dakewe Biotech Company Limited, Shenzhen, China). Standard curves were generated based on the oxLDL content in the sample.

### 2.7. Statistical Analysis

Data are presented as the mean ± SEM. The contractile response of the vascular ring was presented as the percentage relative to the constriction caused by 63.5 mM of K^+^, and the response characteristics were described with the maximum constriction percentage (*E*_max_) and the negative logarithm of the concentration of the receptor agonist causing half *E*_max_ (pEC_50_). Statistical analysis and plotting were performed with Prism 8.0 software. Unpaired Student's *t*-test with Welch's correction was applied to compare two sets of data. One-way analysis of variance (ANOVA) with Dunnett's posttest was used for multiple comparisons. Two-way ANOVA with Bonferroni's posttest was performed to compare the two corresponding data points at each concentration of the two curves. *P* < 0.05 was considered statistically significant.

## 3. Results

### 3.1. The Contractile Response of the Vascular Rings, the Effect of Arterial Endothelium Removal, and the Level of oxLDL

The constriction values of the vascular rings in response to 63.5 mM K^+^ did not show significant differences among the groups (mmLDL, 2.89 ± 0.47 mN; mmLDL + 3-MA, 2.78 ± 0.38 mN; NS, 2.90 ± 0.42 mN; LDL, 2.86 ± 0.44 mN; *P* > 0.05, *n* = 8). Each vascular ring was preconstricted with 20 *μ*M 5-HT followed by dilatation with 10 *μ*M ACh, and the dilatation value was lower than 5% of the preconstriction value, indicating the complete removal of the endothelium. The serum oxLDL concentrations showed a slight difference between the NS group and the mmLDL group, although no significant difference was observed (112.58 ± 6.38 *μ*g/L vs. 111.32 ± 5.84 *μ*g/L; *P* > 0.05, *n* = 8).

### 3.2. Inhibitory Effect of 3-MA on the mmLDL-Induced Upregulated Expression of the ET_A_ Receptor

**Figure 1:**
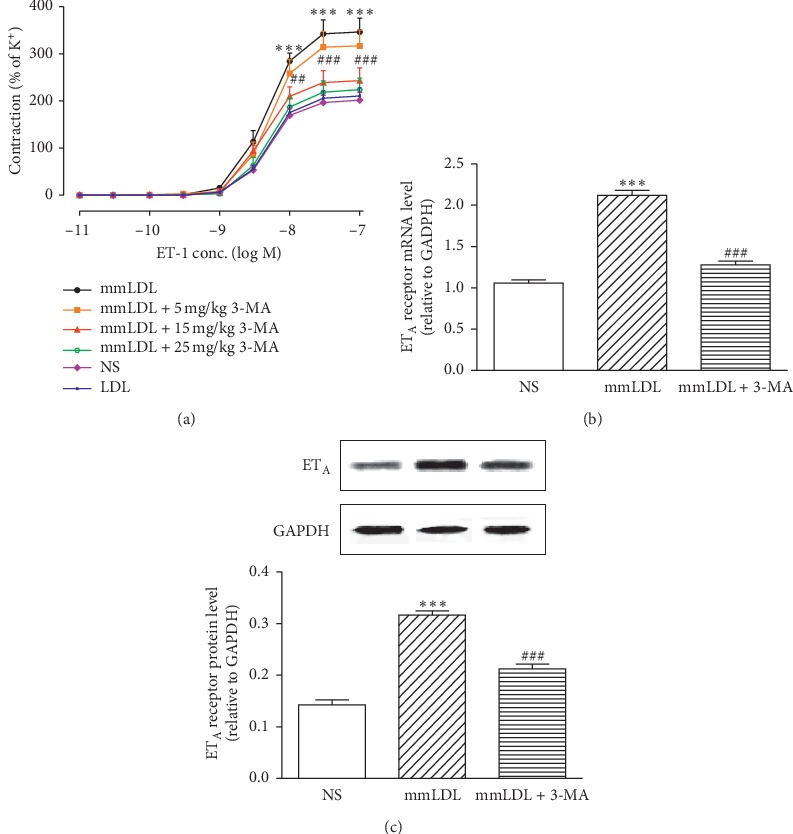
3-MA inhibits the effect of mmLDL on the ET_A_ receptor in VSMCs. (a) 3-MA antagonizes the mmLDL-strengthened contractile curves induced by ET-1 in a dose-dependent manner. (b) The mRNA expression of the ET_A_ receptor (relative to GAPDH; *n* = 6). (c) The protein levels of the ET_A_ receptor (*n* = 4, with each sample as a pool of 2 mesenteric arteries). Data are presented as the mean ± SEM. Statistical analyses were performed using one-way analysis of variance (ANOVA) with Dunnett's posttest and two-way ANOVA with Bonferroni's posttest. ^*∗∗∗*^*P* < 0.001 vs. the NS (normal saline) group. ^##^*P* < 0.01 and ^###^*P* < 0.001 vs. the mmLDL group.

Compared with the NS group, the mmLDL group showed noticeably increased ET_A_ receptor-mediated vasoconstriction, with a noticeable leftward shift of the dose-effect curve. The *E*_max_ value increased from 201.58 ± 16.63% in the NS group to 346.16 ± 29.46% in the mmLDL group (*P* < 0.001), and the pEC_50_ value increased from 8.05 ± 0.05 in the NS group to 9.11 ± 0.09 in the mmLDL group (*P* < 0.01) (Figure 1(a)). mmLDL increased the levels of the ET_A_ receptor: the mRNA expression of the ET_A_ receptor increased from 1.06 ± 0.05 (NS) to 2.12 ± 0.09 (mmLDL) (*P* < 0.001), and the protein level of the ET_A_ receptor increased from 0.14 ± 0.01 (NS) to 0.32 ± 0.01 (mmLDL) (*P* < 0.001) (Figures 1(b) and 1(c)). The supporting data are shown in Supplementary [Supplementary-material supplementary-material-1].

To explore the effect of 3-MA on the experimental outcomes, as well as its optimal dose, the animals were divided into three groups for 3-MA intraperitoneal injections (5, 15, and 25 mg/kg). 3-MA inhibited the mmLDL-induced improvements in vasoconstriction in a dose-dependent manner. Compared with the mmLDL group, the 5 mg/kg group showed a slightly rightward shift of the vasoconstriction curve, with a slightly decreased *E*_max_ value (346.16 ± 29.46% vs. 316.59 ± 33.63%; *P* > 0.05) and a slightly decreased pEC_50_ value (9.11 ± 0.09 vs. 8.82 ± 0.09; *P* > 0.05). Compared with the mmLDL group, the 15 mg/kg group exhibited a noticeable rightward shift of the constriction curve (*E*_max_: 346.16 ± 29.46% vs. 242.85 ± 27.25%, *P* < 0.001; pEC_50_: 9.11 ± 0.09 vs. 8.22 ± 0.09, *P* < 0.05). Although the constriction curve of the 25 mg/kg group showed a slightly rightward shift compared with that of the 15 mg/kg group, no significant differences were observed (in the 25 mg/kg group, the *E*_max_ and pEC_50_ values were 224.07 ± 24.23% and 8.05 ± 0.06, respectively; Figure 1(a)). Therefore, 15 mg/kg was used as the optimal dose of 3-MA to further explore the role of Class III PI3K/Beclin-1 in the upregulation of the ET_A_ receptor induced by mmLDL. 3-MA at 15 mg/kg significantly inhibited the strengthening effect of mmLDL on the ET_A_ receptor: the mRNA expression and protein level of the ET_A_ receptor decreased to 1.28 ± 0.06 (*P* < 0.001) and 0.21 ± 0.01 (*P* < 0.001) (Figures 1(b) and 1(c)). The caudal vein injection of LDL at 1 mg/kg did not markedly affect the ET_A_ receptor-mediated constriction curve (Figure 1(a)).

### 3.3. Inhibitory Effect of 3-MA on the mmLDL-Induced Effects on Class III PI3K, Beclin-1, LC3 II/I, and p62 Proteins

**Figure 2:**
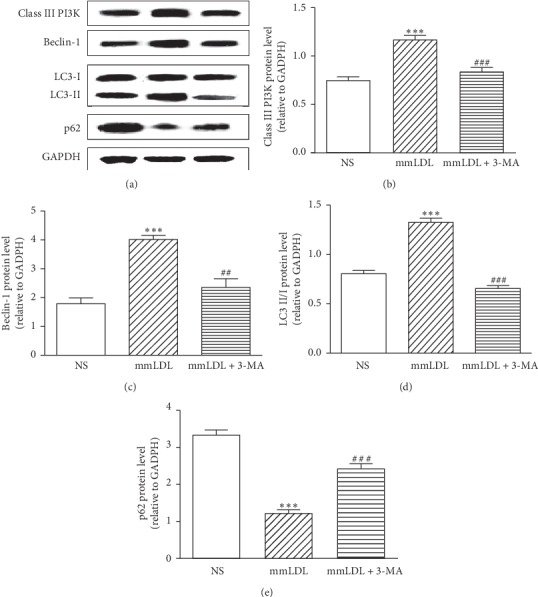
Western blot analysis showing that 3-MA inhibits the effect of mmLDL on the protein levels of Class III PI3K, Beclin-1, LC3 II/I, and p62. (a) The protein levels of class III PI3K. (b) The protein levels of Beclin-1. (c) The protein levels of LC3 II/I. (d) The protein levels of p62. All values are expressed as the mean ± SEM. *n* = 4, with each sample as a pool of 2 mesenteric arteries. Statistical analysis was performed using one-way ANOVA with Dunnett's post-test. ^*∗∗*^*P* < 0.01 and ^*∗∗∗*^*P* < 0.001 vs. the NS (normal saline) group. ^##^*P* < 0.01 and ^###^*P* < 0.001 vs. the mmLDL group.

After caudal vein injection of mmLDL, the protein levels of Class III PI3K, Beclin-1, and LC3 II/I increased from 0.75 ± 0.04, 1.79 ± 0.24, and 0.80 ± 0.04 to 1.17 ± 0.04, 4.01 ± 0.17, and 1.33 ± 0.05 (*P* < 0.001, *P* < 0.001, and *P* < 0.001), respectively, and the p62 protein level decreased from 3.35 ± 0.10 (NS) to 1.23 ± 0.09 (*P* < 0.001). 3-MA significantly decreased the mmLDL-induced increase in the protein levels of Class III PI3K, Beclin-1, and LC3 II/I to 0.84 ± 0.05, 2.36 ± 0.34, and 0.66 ± 0.03 (*P* < 0.001, *P* < 0.01, and *P* < 0.001), respectively, and increased the mmLDL-induced decrease in p62 protein level to 2.44 ± 0.13 (*P* < 0.001), indicating that 3-MA inhibited the autophagy activation caused by mmLDL (Figures 2(a)–2(d); Supplementary [Supplementary-material supplementary-material-1]).

### 3.4. Inhibitory Effect of 3-MA on the mmLDL-Induced Effect on p-NF-*κ*B Protein

**Figure 3:**
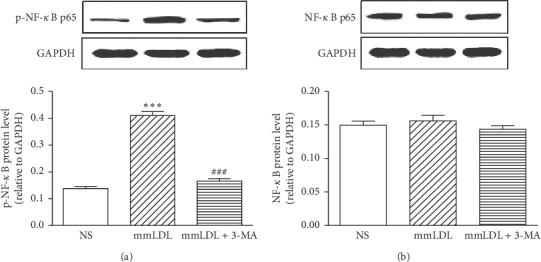
Western blot analysis showing the effect of mmLDL and 3-MA on the protein levels of p-NF-*κ*B and NF-*κ*B. (a) The protein levels of p-NF-*κ*B. (b) The protein levels of NF-*κ*B. All values are expressed as the mean ± SEM. *n* = 4, with each sample as a pool of 2 mesenteric arteries. Statistical analysis was performed using one-way ANOVA with Dunnett's post-test. ^*∗∗∗*^*P* < 0.001 vs. the NS (normal saline) group. ^###^*P* < 0.001 vs. the mmLDL group.

The mmLDL group showed an increased level of arteriomesenteric p-NF-*κ*B protein, which increased from 0.14 ± 0.01 (NS) to 0.41 ± 0.02 (*P* < 0.001). Compared with the mmLDL group, the 3-MA group showed a significantly decreased p-NF-*κ*B level (0.17 ± 0.01; *P* < 0.001; Figure 3(a)). The NF-*κ*B protein levels in the NS, mmLDL, and 3-MA + mmLDL groups were 0.15 ± 0.04, 1.16 ± 0.01, and 0.14 ± 0.01, respectively (all *P* > 0.05; Figure 3(b)). The supporting data for this analysis are shown in Supplementary [Supplementary-material supplementary-material-1].

## 4. Discussion

mmLDL is a risk factor for cardiovascular diseases, which causes damage to endothelial cells and induces the production of foam cells. However, the pathogenic mechanisms of mmLDL remain unclear. For the first time, this study indicated that caudal vein injection of mmLDL could upregulate the ET_A_ receptor level in mouse arteriomesenteric smooth cells to strengthen ET_A_ receptor-mediated vasoconstriction. Furthermore, this study discovered that the Class III PI3K/Beclin-1 autophagy pathway participated in the regulation of the arteriomesenteric ET_A_ receptor level in mice *in vivo* for the first time.

The increased ET_A_ receptor level plays an important role in the pathogenesis of multiple cardiovascular diseases, such as hypertension [16, 17], pulmonary hypertension [18, 19], dilated cardiomyopathy, and microvascular dysfunction [20, 21]. ET_A_ receptor knockout can reverse or reduce the aging-induced downregulation of autophagy markers [6]. In the myocardial cell protection mediated by ET_A_ receptor knockout or the application of ET_A_ receptor antagonists, autophagy plays a critical role [22]. 3-MA eliminates the effect on cardiovascular proteins induced by the ET_A_ receptor antagonist BQ123 and inhibits the formation of autophagosomes and the degradation of lysosomes; however, both of these effects can be reversed by ET_A_ receptor knockout [6]. In this study, 3-MA inhibited the mmLDL-induced upregulation of the ET_A_ receptor, which also indicated the important roles of autophagy in the regulation and protection of the cardiovascular system.

Autophagy is closely associated with cardiovascular diseases: physiological conditions, such as nutritional deficiency, can induce the autophagy of isolated myocardial cells, vascular epithelial cells, and VSMCs [23]. Under these conditions, autophagy can improve the accumulation and degradation of organelles and proteins that are lower than normal levels [24]. Under cardiovascular stress, such as ischemia reperfusion and heart failure, autophagy can be activated. However, whether autophagy is beneficial or harmful under these conditions of stress has not been definitively determined [25, 26].

VSMC autophagy has an important role in vascular disease development [27]. During autophagy, microvessel-associated LC3-I transforms from the cytoplasmic form to the LC3 phosphatidylethanolamine-binding form (LC3-II) to promote the formation of autophagosomes, thereby maintaining their growth. The formation of LC3-II is normally used as an indicator of autophagy activation [28]. In this study, LC3-II showed a relatively high protein level in VSMCs after mmLDL injection, which indicated that mmLDL increased the autophagy level of VSMCs. In contrast, 3-MA inhibited the effect of mmLDL on autophagy activation and downregulated the protein ratio of LC3-II/LC3-I, which indicated that 3-MA reduced the autophagy level of VSMCs.

p62 (a scaffold protein) and LC3-II interact with polyubiquitinated proteins, which causes the degradation of the latter. The activation of the autophagy process can decrease the content of p62. Therefore, the p62 level can indicate whether autophagy is complete [29]. In this study, compared with NS, mmLDL significantly decreased the p62 level in VSMCs *in vivo*, which indicated that mmLDL activated the autophagy process.

Beclin-1 is associated with the formation of autophagic vacuoles, and its level has been considered to be a reliable marker to detect autophagy flux [30]. In this study, compared with NS, mmLDL upregulated the protein levels of Beclin-1 and Class III PI3K, which indicated that mmLDL promoted the formation of the autophagic vacuoles in VSMCs. mmLDL not only increased the ET_A_ receptor level in VSMCs but also induced the autophagy of VSMCs.

The NF-*κ*B pathway participates in the upregulation of the ET_B_ receptor in VSMCs in vitro [31]. As a stress-sensitive transcription factor, NF-*κ*B possesses remarkable proliferation and antiapoptotic and proinflammatory effects. The activation of NF-*κ*B upregulates Beclin-1 and promotes autophagy [32]. Beclin-1/Atg 6 and Class III PI3K/Vps 34 act jointly, recruiting autophagy activators and inhibitors to adjust the formation of autophagosomes in a specific manner [33]. LC3 binds to phosphatidylethanolamine, which allows it to integrate with the membrane of autophagosomes for autophagosome formation [34]. In this study, mmLDL upregulated the protein phosphorylation level of NF-*κ*B, whereas 3-MA downregulated the mmLDL-induced increase in NF-*κ*B phosphorylation, which indicated that Class III PI3K/Beclin-1 participated in NF-*κ*B activation. For the first time, this study elucidated that the NF-*κ*B pathway participated in the mmLDL-induced upregulation of the ET_A_ receptor in VSMCs.

mmLDL upregulates the protein levels of Class III PI3K, Beclin-1, and LC3 II/I in arteriomesenteric VSMCs and downregulates the protein level of p62; that is, mmLDL activates the autophagy of VSMCs and upregulates the protein and mRNA expression of the ET_A_ receptor to strengthen the ET_A_ receptor-mediated vasoconstriction effect. In addition, the autophagy activation of VSMCs affects the upregulation of the ET_A_ receptor. 3-MA downregulates the increased autophagy level induced by mmLDL and inhibits the upregulation of the ET_A_ receptor caused by mmLDL. Therefore, mmLDL activates the autophagy of arteriomesenteric VSMCs *in vivo* to upregulate the ET_A_ receptor level.

## Data Availability

The data used to support the findings of this study are included within the article and the supplementary files.

## Supplementary Materials

Supplementary Materials Supplementary File 1: inhibitory effect of 3-MA on the mmLDL-induced upregulated expression of the ETA receptor. Supplementary File 2: inhibitory effect of 3-MA on the mmLDL-induced effects on Class III PI3K, Beclin-1, LC3 II/I, and p62 proteins. Supplementary File 3: inhibitory effect of 3-MA on the mmLDL-induced effect on p-NF-*κ*B protein.
